# Identification of cuproptosis‐related lncRNAs for prognosis and immunotherapy in glioma

**DOI:** 10.1111/jcmm.17603

**Published:** 2022-11-01

**Authors:** Lin Wang, Yunqian Li, Yubo Wang, Jia Li, Yajuan Sun, Jiajun Chen, Ziqian Wang

**Affiliations:** ^1^ Department of Neurology China‐Japan Union Hospital of Jilin University Changchun China; ^2^ Department of Neurosurgical Oncology The First Hospital of Jilin University Changchun China

**Keywords:** cuproptosis, glioma, lncRNA, prognosis, risk score

## Abstract

Glioma is a highly invasive primary brain tumour, making it challenging to accurately predict prognosis for glioma patients. Cuproptosis is a recently discovered cell death attracting significant attention in the tumour field. Whether cuproptosis‐related genes have prognostic predictive value has not been clarified. In this study, uni‐/multi‐variate Cox and Lasso regression analyses were applied to construct a risk model based on cuproptosis‐related lncRNAs using TCGA and CGGA cohorts. A nomogram was constructed to quantify individual risk, including clinical and genic characteristics and risk. GO and KEGG analyses were used to define functional enrichment of DEGs. Tumour mutation burden (TMB) and immune checkpoint analyses were performed to evaluate potential responses to ICI therapy. Ten prognostic lncRNAs were obtained from Cox regression. Based on the median risk score, patients were divided into high‐ and low‐risk groups. Either for grade 2–3 or for grade 4, glioma patients with high‐risk exhibited significant poorer prognoses. The risk was an independent risk factor associated with overall survival. The high‐risk group was functionally associated with immune responses and cancer‐related pathways. The high‐risk group was associated with higher TMB scores. The expression levels of many immune checkpoints in the high‐risk group were significantly higher than those in the low‐risk group. Differentiated immune pathways were primarily enriched in the IFN response, immune checkpoint and T‐cell co‐stimulation pathways. In conclusion, we established a risk model based on cuproptosis‐related lncRNAs showing excellent prognostic prediction ability but also indicating the immuno‐microenvironment status of glioma.

## 1 INTRODUCTION

Glioma is the most prevalent primary malignant brain tumour including lower‐grade glioma (LGG) and glioblastoma (GBM).^1^ GBM has a poor prognosis with a median survival of 14 months and exhibits resistance to therapy.^2^ The clinical prognosis of LGG is highly variable, although early diagnosis and risk evaluation can help improve the prognosis.^3^ Nonetheless, the rapid progression and the high heterogeneity of glioma make precise prognostic prediction challenging. In addition, a large number of clinical trials have been conducted targeting biomarkers for glioma treatment, but few have been successful. Therefore, it is urgent to identify new biomarkers to establish a risk prediction model for glioma.^4^

Copper is considered a double‐edged sword in metabolic pathways for all organisms. Copper is an essential cofactor for many enzymes; however, excess intracellular copper is toxic and can induce cell death.^5^ Cuproptosis is a recently discovered copper‐mediated cell death pathway, which is distinct from known cell death pathways including apoptosis, necroptosis and ferroptosis.^6^, ^7^, ^8^ Unbalanced copper homeostasis results in irreversible damage to tumour cells. Both copper chelators and copper ionophores show anticancer properties.^9^, ^10^, ^11^, ^12^ However, the role that cuproptosis plays in glioma has not been clarified. It is necessary to study whether cuproptosis‐related genes have prognostic predictive value.

Long‐non‐coding RNAs (lncRNAs) are a class of non‐coding RNAs with the length >200 nt. LncRNAs play an important functional role in regulating almost all the biological processes, including cell proliferation, cell cycle, cell invasion and immune response. It has been generally revealed that numerous lncRNAs are expressed aberrantly in many cancers, and some lncRNAs appear to be cancer‐specific.^13^ For example, many lncRNAs have been found involved in glioma progression such as MALAT1, XIST, HOXD‐AS1, CCAT1, LOC728196 and SNHG20.^14^, ^15^ In addition, most lncRNAs are stable and detectable in body fluids, making lncRNAs a promising choice for non‐invasive biomarkers and therapeutic targets for cancers. The functional roles of lncRNAs in pathology, diagnosis, prognosis and therapy of various cancers need to be determined urgently. Among the alternative methods, computational models are anticipated to be an effective way, which predict potential LncRNA‐Disease Associations (LRLSLDA) on a large scale.^16^ LRLSLDA is an approach ranking disease–lncRNA candidates for all the diseases simultaneously. In a study by Chen et al, a computational model of Laplacian Regularized Least Squares for LRLSLDA was developed.^17^ Regression models including Cox, Lasso and Ridge are some widely used approaches well suited for constructing computational models when there are large numbers of features.^18^ Many studies have highlighted the power of lncRNA‐based computational models to predict glioma prognosis and tumour response to therapy. The pathways involved in these lncRNAs include immune, autophagy, ferroptosis and pyroptosis.^19^, ^20^, ^21^, ^22^ However, a prognosis predictive model based on cuproptosis‐related lncRNAs for glioma has not been reported.

In this study, 10 cuproptosis‐related lncRNAs were identified to construct a risk model for the prognosis prediction in glioma patients.^5^ Our work firstly revealed that cuproptosis‐related lncRNAs possessed powerful potential as prognostic markers and therapeutic targets for glioma.

## 2 METHODS

### 2.1 Data acquisition and processing

mRNA expression, lncRNA expression, simple nucleotide variation data and clinical information were downloaded from LGG and GBM datasets from different brain sites in the Cancer Genome Atlas dataset (TCGA, https://portal.gdc.cancer.gov/). The sample size for transcriptome data (mRNA and lncRNA) was 688, clinical data was from 1112 patients, and the number of included simple nucleotide variants was 991. The sample size for the intersection of transcriptome data, simple nucleotide variation data and clinical data was 653. Furthermore, samples were downloaded from the Chinese Glioma Genome Atlas (CGGA, http://cgga.org.cn/), mRNAseq_693 and mRNAseq_325 projects.^23^ Patients with missing clinical information were excluded. Clinical information is summarized in Table S1. The raw data of the RNA‐seq cohorts were transformed from the fragments per kilobase of transcript per million (FPKM) format to the transcripts per kilobase million (TPM) format. Data from TCGA dataset (653 patients) were considered the training group, and the CGGA dataset including 1018 patients was considered the testing group.

From the study by Tsvetkov et al., 10 cuproptosis genes were defined (resistance genes: FDX1, LIAS, LIPT1, DLD, DLAT, PDHA1 and PDHB; sensitizer genes: MTF1, GLS and CDKN2A). LncRNAs associated with cuproptosis genes were obtained by Pearson's correlation analysis using the package ‘limma’ in R. A lncRNA was identified to be associated with the cuprotosis gene when at least a moderate correlation (*r* > 0.5) between them and the Bonferroni adjusted *p*‐value <0.05.^24^ A total of 486 lncRNAs associated with cuproptosis genes were selected.

### 2.2 Construction of the prognostic model

In order to adjust for batch effects, lncRNA expression was normalized using the ‘sva’ package in R. Thirty‐three of 486 cuprotosis‐related lncRNAs were identified with the stepwise univariate Cox proportional hazards regression model (Bonferroni adjusted *p*‐value <0.0001). Afterwards, the aLASSO (least absolute shrinkage and selection operator) regression was constructed using the lncRNAs extracted from the previous univariate Cox proportional hazards regression model to further refine the model and increase the robustness of the model. Lambda.min was 0.02757539 and was used to identify 15 cuprotosis‐related lncRNAs. Furthermore, multivariate Cox regression analysis ultimately identified 10 cuproptosis‐related lncRNAs to establish a more robust prognostic risk model. The risk score was the sum of the product of lncRNA expression levels and coefficients. Patients were divided into high‐risk groups (risk value > median) and low‐risk groups (risk value < median) according to the median risk score. Risk score = (0.189 × DLGAP1‐AS1) + (0.238 × LINC00665) + (−0.368 × MIR4500HG) + (−0.371 × NNT‐AS1) + (0.668 × PAXIP1‐AS2) + (0.192 × PITPNA‐AS1) + (−0.204 × SNAI3‐AS1) + (−0.263 × STX18‐AS1) + (−0.297 × TTC28‐AS1) + (0.612 × UBA6‐AS1). Kaplan–Meier survival analysis and the log‐rank test were employed to compare OS differences between high‐ and low‐risk groups.

### 2.3 Predictive nomogram and receiver operating characteristic curve

Combining multiple risk factors, we used nomograms to quantify individual risk in the clinical setting. Independent predictors identified by multivariate Cox regression were integrated into the nomogram. The ‘rms’ R package was used to construct the predictive nomogram and the corresponding calibration curves. The closer the calibration curve was to the diagonal, the better the prognostic predictive performance of the nomogram. The receiver operating characteristic (ROC) curve was used to assess the accuracy and sensitivity of the model based on the prognostic lncRNA signature.

### 2.4 Prediction of independent prognostic factors and target genes

The relationship between overall prognosis and OS time was analysed by univariate Cox regression, as well as clinical variables (including age, gender and the World Health Organization glioma classification). We considered a variable as an independent prognostic factor only if the *p*‐values in the uni‐ and multivariate Cox regression were both <0.05.

The differential expression of mRNAs between the high‐ and low‐risk groups was compared using the screening criteria of *p* < 0.05 and |log2 fold change (FC)| > 2. The Kyoto Encyclopedia of Genes and Genomes (KEGG) pathways and gene ontology (GO) enrichment analysis were performed using the R package ‘clusterProfiler’. To analyse infiltrating scores between high‐ and low‐risk groups, scores indicating immune cell infiltration and the involvement of immune‐related pathways were calculated for each sample by single sample gene set enrichment analysis (ssGSEA) using the R package ‘gsva’. To determine differences in gene alteration between the high‐ and low‐risk groups, we mapped the mutation profiles of the first 15 genes using the R package ‘Maftools’.^25^

### 2.5 Tumour mutation burden analysis and immune checkpoint analysis

Tumour mutation burden (TMB) was calculated as the total of nonsynonymous variants in each sample including deletion, splice site, nonsense mutation, missense mutation and insertion. TMB was considered as both a binary variable (TMB‐L vs. TMB‐H defined as ≥17 mut/Mb) and a continuous variable of TMB magnitude. The threshold to define TMB‐H was established by comparing TMB with MSI by fragment analysis in CRC cases. The ggplot2 package was used to visualize the relationship between the TMB and gene mutations. TMB differences between the high‐ and low‐risk groups were analysed. The expression levels of immune checkpoints were compared between the high‐ and low‐risk groups in the TCGA cohort.

### 2.6 Analysis

All statistical tests were performed in R 4.2.0 and the appropriate toolboxes. Independent prognostic factors were determined by univariate, LASSO regression and multivariate Cox regression analysis. If the data were normally distributed, the *t*‐test was used; otherwise, the *u*‐test was applied. Dichotomic data were tested using the chi‐square test.

## 3 RESULTS

### 3.1 Identification of 10 cuproptosis‐related lncRNAs with prognostic significance

**FIGURE 1:**
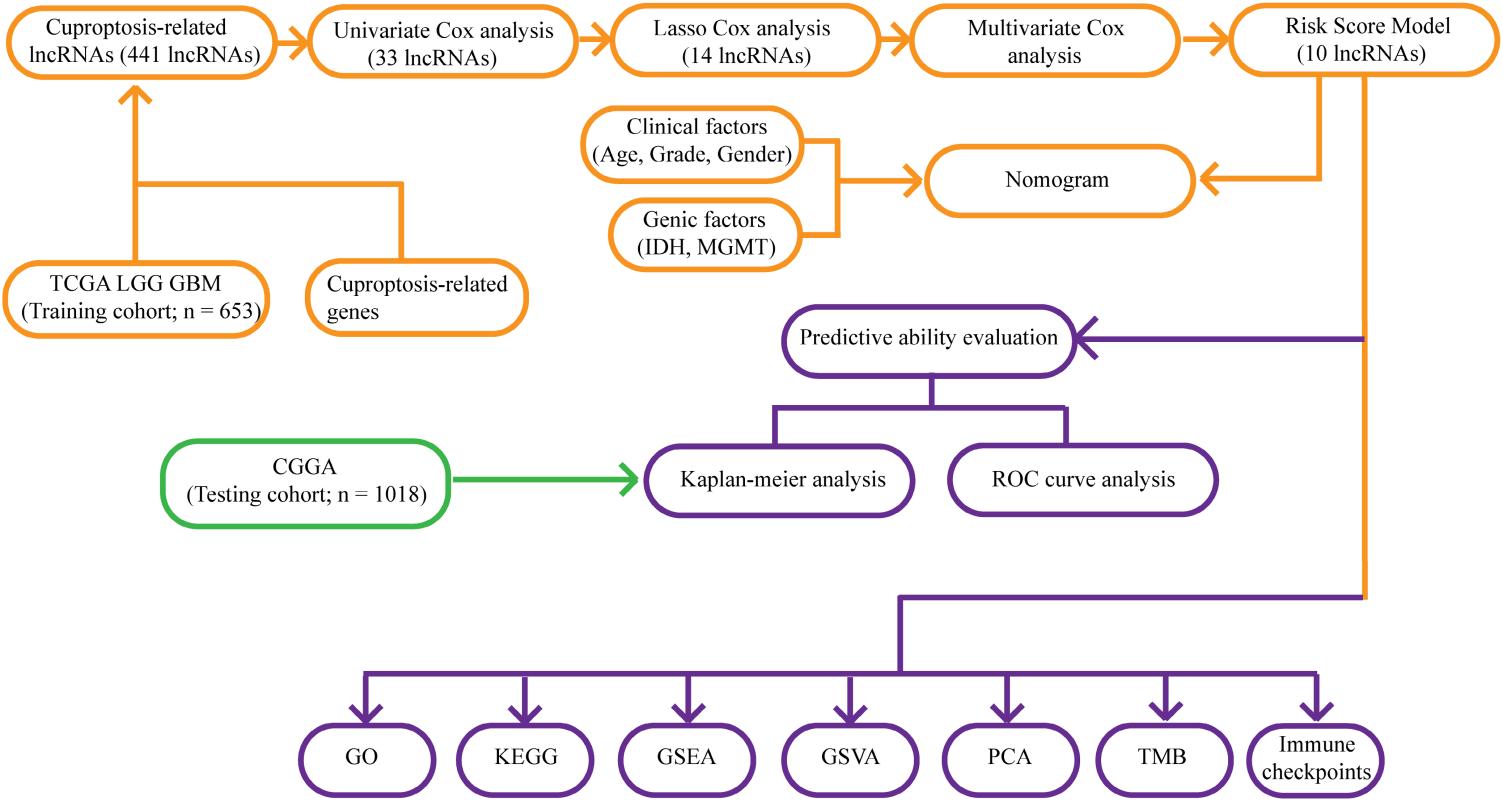
Workflow of this study.

**FIGURE 2:**
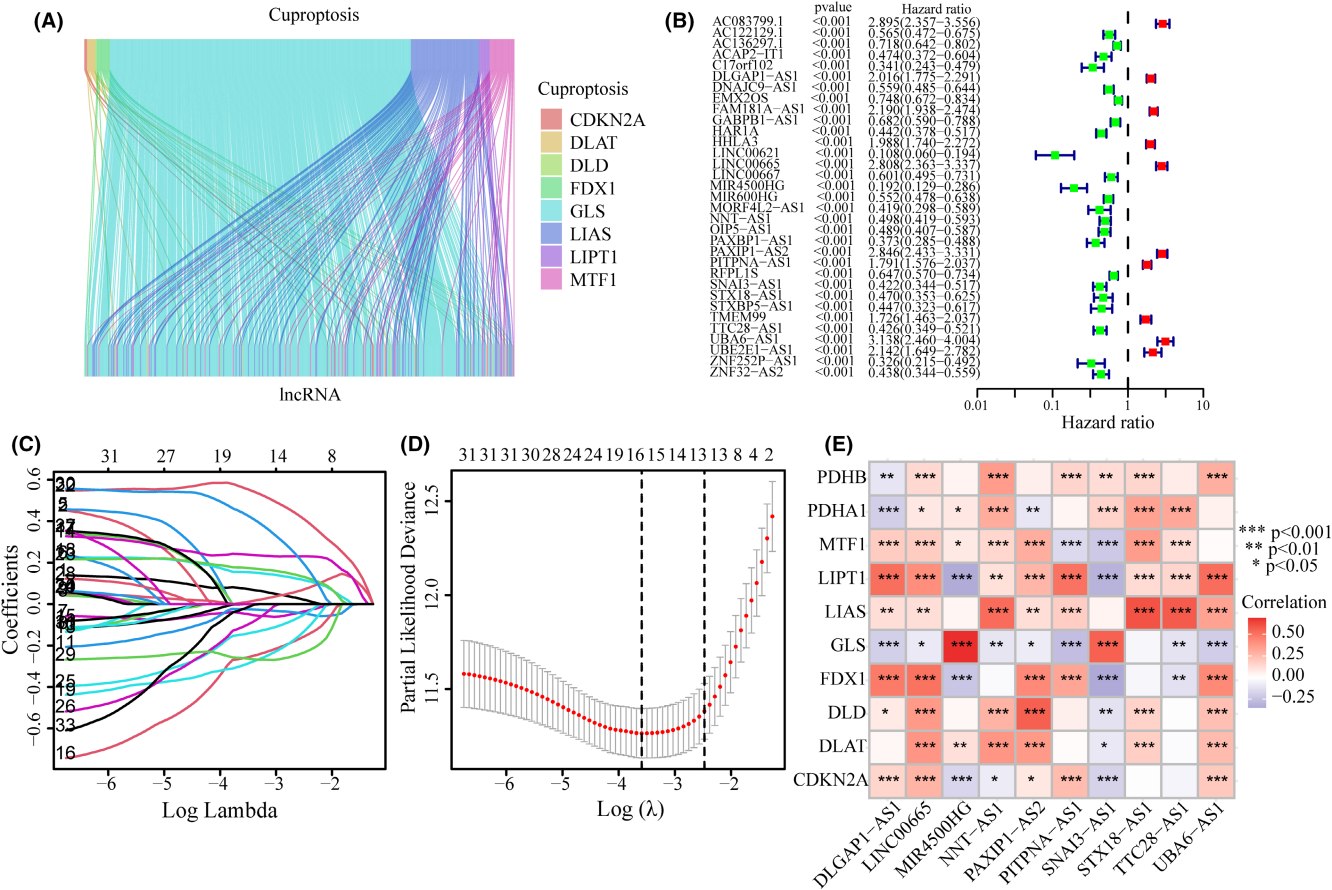
Identification of CRlncRNAs with prognostic significance. (A) A Sankey diagram in TCGA cohort showed the relationship between lncRNAs and 10 cuproptosis‐related mRNAs. (B) Univariate Cox regression analysis screened CRlncRNAs. (C, D) Lasso regression analysis screened CRlncRNAs. (E) After multivariate Cox regression analysis, 10 CRlncRNAs were screened. Co‐expression analysis on cuproptosis‐related mRNAs and these CRlncRNAs was performed.

The workflow of this study was shown in Figure 1. The expression data of a total of 16,876 lncRNAs associated with glioma were extracted from TCGA database. Pearson's correlation analysis was conducted to evaluate the co‐expression relationship between the lncRNAs and 10 cuproptosis‐related genes. A Sankey diagram showed the connection degree between the 10 cuproptosis‐related mRNAs and the 486 lncRNAs (Figure 2A). To identify cuproptosis‐related lncRNAs (CRlncRNAs) associated with prognosis, univariate Cox hazard analysis was used and 35 CRlncRNAs were identified from 486 lncRNAs with |*R*| > 0.5 and Bonferroni adjusted *p* < 0.0001 (Figure 2B). Subsequently, LASSO Cox analysis was performed and 15 lncRNAs were selected (Figure 2C,D). Multivariate Cox analysis further determined 10 CRlncRNAs with prognostic significance. The co‐expression relationship between these 10 lncRNAs and 10 cuproptosis‐related mRNAs was shown in Figure 2E.

### 3.2 Risk score model based on 10 CRlncRNAs

A proportional hazard model consisting of 10 CRlncRNAs was constructed using multivariate Cox analysis. Risk score = (0.189 × DLGAP1‐AS1 expression level) + (0.238 × LINC00665 expression level) + (−0.368 × MIR4500HG expression level) + (−0.371 × NNT‐AS1 expression level) + (0.668 × PAXIP1‐AS2 expression level) + (0.192 × PITPNA‐AS1 expression level) + (−0.204 × SNAI3‐AS1 expression level) + (−0.263 × STX18‐AS1 expression level) + (−0.297 × TTC28‐AS1 expression level) + (0.612 × UBA6‐AS1 expression level).

### 3.3 Evaluation of the CRlncRNA‐based prediction model for glioma

**FIGURE 3:**
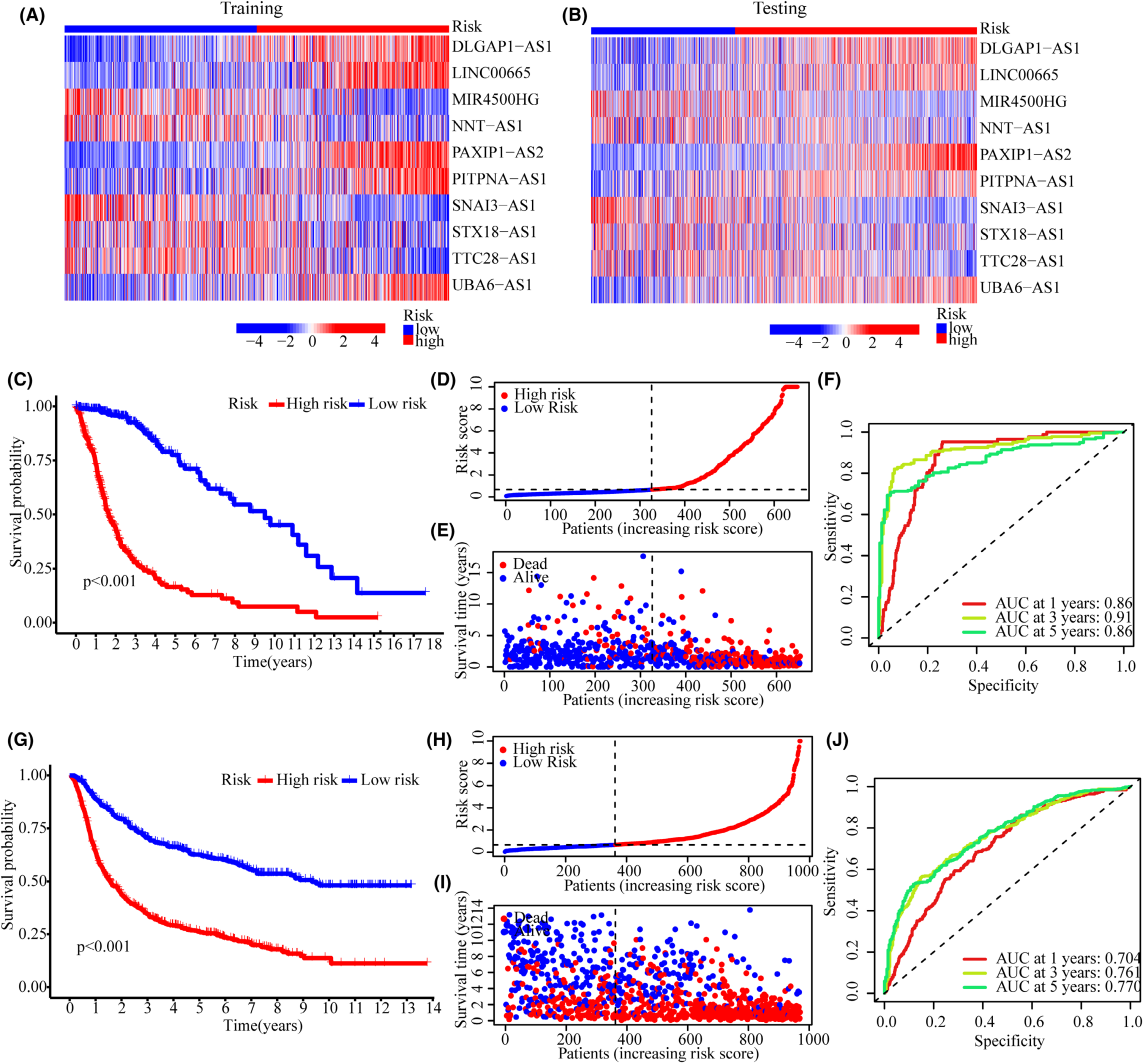
Risk model evaluation. (A, B) Heatmaps showing 10 CRlncRNA expression patterns in low‐ and high‐risk subgroups of the Training (A) and Testing cohorts (B, C) Kaplan–Meier analysis in the Training group. (D, E) The risk score and corresponding survival status of the Training group. (F) ROC curve analysis of this model for predictive accuracy in TCGA cohort. (G) Kaplan–Meier analysis in the Testing group. (H, I) The risk score and corresponding survival status of the Testing group. (J) ROC curve analysis of this model for predictive accuracy in CGGA cohort.

Glioma patients were divided into low‐ and high‐risk groups according to the median risk score. For the Training and Testing groups, the expression patterns of 10 CRlncRNA in the low‐ and high‐risk subgroups were shown in the heatmaps in Figure 3A,B. The CRlncRNA expression patterns in the Training and Testing group were similar. For the training group, the survival probability of the high‐risk group was significantly lower than that of the low‐risk group (Figure 3C; *p* < 0.001). The risk score and survival status of the Training group showed that the mortality rate increased with higher scores (Figure 3D,E). ROC curves indicated a good accuracy of this model for predicting survival in TCGA (AUC_risk_ at 1 year = 0.874, AUC_risk_ at 3 year = 0.930, AUC_risk_ at 5 year = 0.890; Figure 3F). For the Testing group, the survival probability of the high‐risk group was lower than that of the low‐risk group (Figure 3G; *p* < 0.001). Further, the mortality rate was higher in the high‐risk subgroup than in the low‐risk subgroup (Figure 3H,I). The good accuracy of the model for predicting survival in CGGA is shown in Figure 3J.

### 3.4 The CRlncRNA‐based model is an independent prognostic factor for glioma patients

**FIGURE 4:**
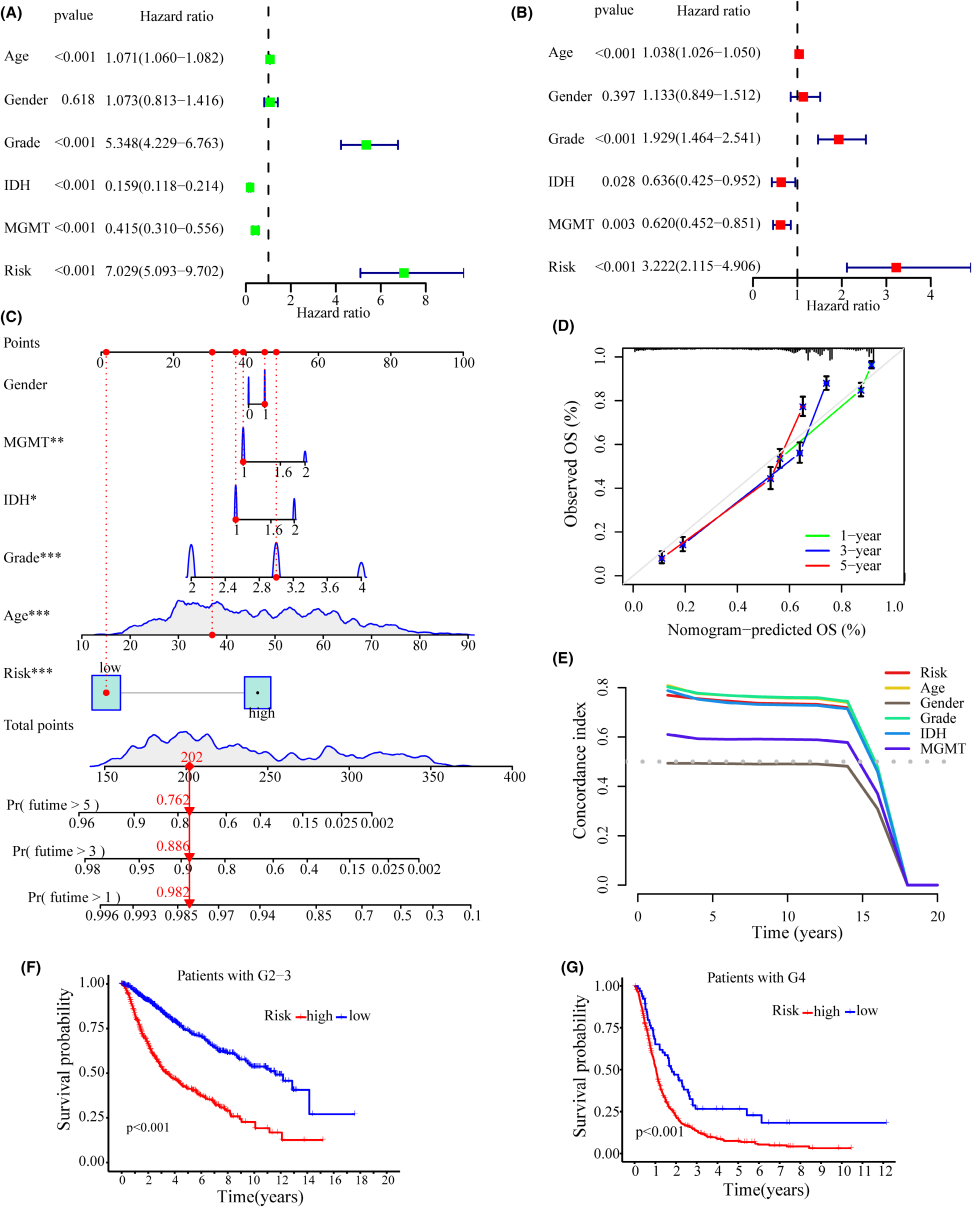
Analysis of independent prognostic factors. (A, B) Forest plots showing univariate Cox (A) and multifactor Cox (B) regression analyses of training cohort. (C) The nomogram based on risk and clinical factors to predict the 1‐, 3‐ and 5‐year survival of glioma patients. (D) The calibration plot for the nomogram. (E) Concordance index to assess the predictive ability of risk score. (F, G) Kaplan–Meier analyses on the grade 2–3 (F) and grade 4 (G) of glioma patients, respectively.

Uni‐ and multi‐variate Cox regression analyses were used to determine whether the CRlncRNA‐based model is an independent prognostic factor in glioma (Figure 4A,B). Multivariate Cox analysis showed that age (HR = 1.038, 1.026–1.050, *p* < 0.001), grade (HR = 1.929, 1.464–2.541, *p* < 0.001), IDH mutation (HR = 0.636, 0.425–0.952, *p* = 0.028), MGMT methylation (HR = 0.620, 0.452–0.851, *p* = 0.003) and the risk of the CRlncRNA‐based model (HR = 3.222, 2.115–4.906, *p* < 0.001) were independent prognostic factors (Figure 4B). Subsequently, gender, age, grade, IDH mutation, MGMT methylation and risk were included to build a nomogram predicting 1‐, 3‐ and 5‐year survival of glioma patients (Figure 4C). The calibration plot indicated a good prediction accuracy of the nomogram on 1‐year survival (Figure 4D). Then, the concordance index was measured to estimate prediction accuracy. The results of the Concordance Index showed good precision of the predictions by grade, age and especially risk score (Figure 4E). To investigate the prognostic value of the CRlncRNA‐based model in different grades of glioma, survival analyses were performed on grade 2–3 and grade 4 glioma patients, respectively (Figure 4F,G). The results of the survival analyses showed that in both grade 2–3 and grade 4 glioma patients, the high‐risk group had a significantly poorer prognosis than the low‐risk group (grade 2–3, *p* < 0.001; grade 4, *p* < 0.001; Figure 4F,G).

### 3.5 Principal component analysis and functional enrichment analysis

**FIGURE 5:**
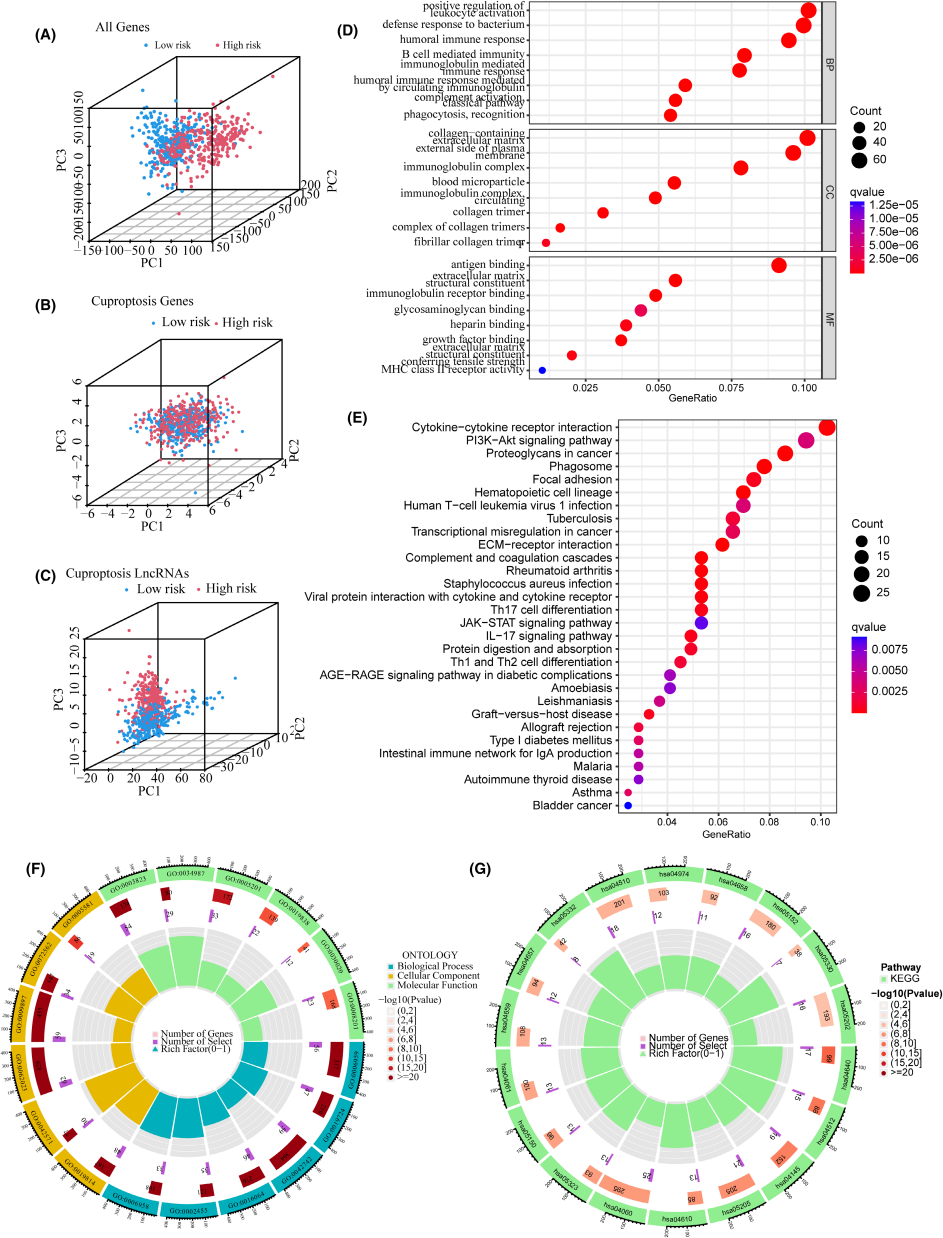
Principal component analysis (PCA) and functional enrichment analysis. (A–C) PCA based on the whole genome (A), cuproptosis‐related mRNAs (B), and cuproptosis‐related lncRNAs (C). (D, F) GO analysis for DEGs between the low‐ and high‐risk groups in TCGA cohort. (E, G) KEGG analysis for DEGs between the low‐ and high‐risk groups in TCGA cohort.

Based on the whole genome, cuproptosis‐related mRNAs, cuproptosis‐related lncRNAs and the 10 lncRNAs for the CRlncRNA‐based model, principal component analysis (PCA) was used to visualize the differences between the low‐ and high‐risk groups. The results suggested that compared with other gene sets, the low‐ and high‐risk groups stratified by cuproptosis‐related lncRNAs tended to have a more scattered distribution (Figure 5A–C). A total of 652 DEGs between the low‐ and high‐risk groups were identified in the TCGA cohort. The results of the GO analysis showed that DEGs functionally enriched in humoral immune response, B‐cell‐mediated immunity, immunoglobulin‐mediated immune response, complement activation, phagocytosis and recognition (Figure 5D,F). The results of the KEGG analysis revealed enriched pathways primarily in cytokine receptor interaction, the PI3K Akt signalling pathway, proteoglycans in cancer and dysregulated transcriptional activity in cancer (Figure 5E,G).

### 3.6 TMB and immune checkpoint analysis

**FIGURE 6:**
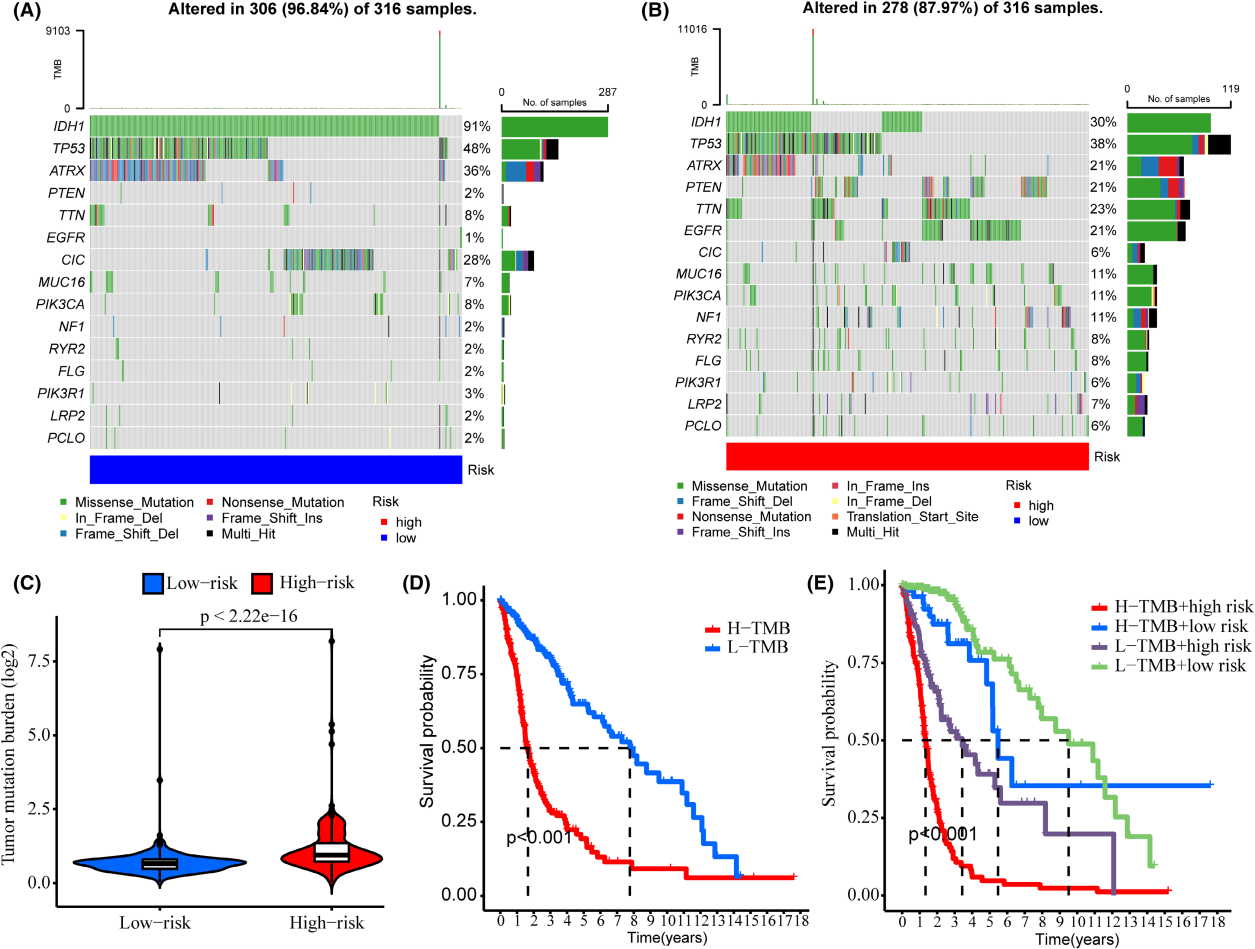
TMB analysis. (A, B) Waterfall plots showing the top 15 genes with the highest alteration frequencies of the low‐ (A) and high‐risk (B) groups. (C) TMB scores of the low‐ and high‐risk groups. (D) Kaplan–Meier analysis showing the overall survival of low‐ and high‐TMB groups. (E) Kaplan–Meier analysis showing the overall survival of low‐ and high‐risk subgroups in low‐ and high‐TMB groups, respectively.

**FIGURE 7:**
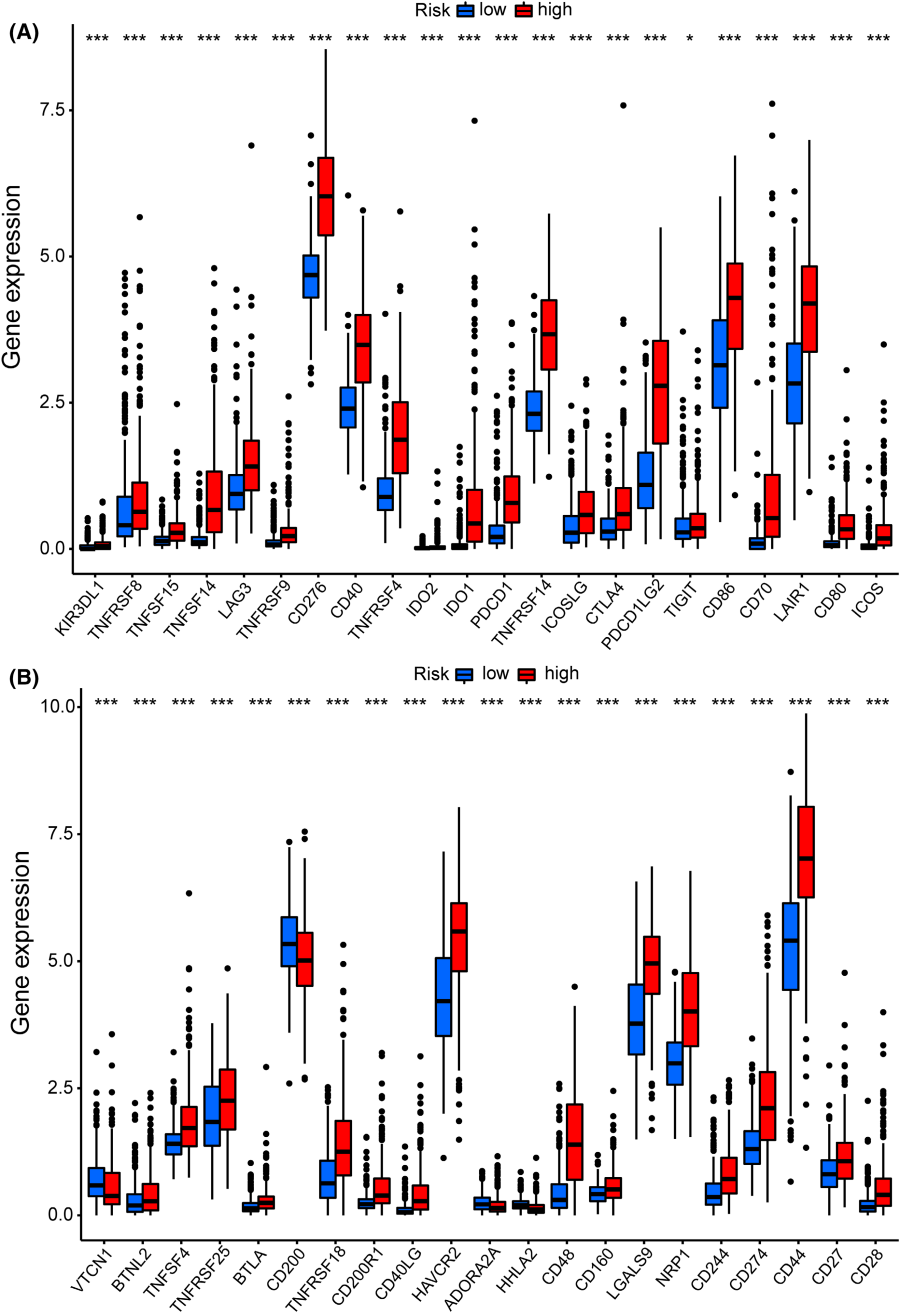
Immune checkpoint analysis in the TCGA cohort. (A, B) Expression of immune checkpoints between the high‐ and low‐risk groups in the TCGA cohort.

**FIGURE 8:**
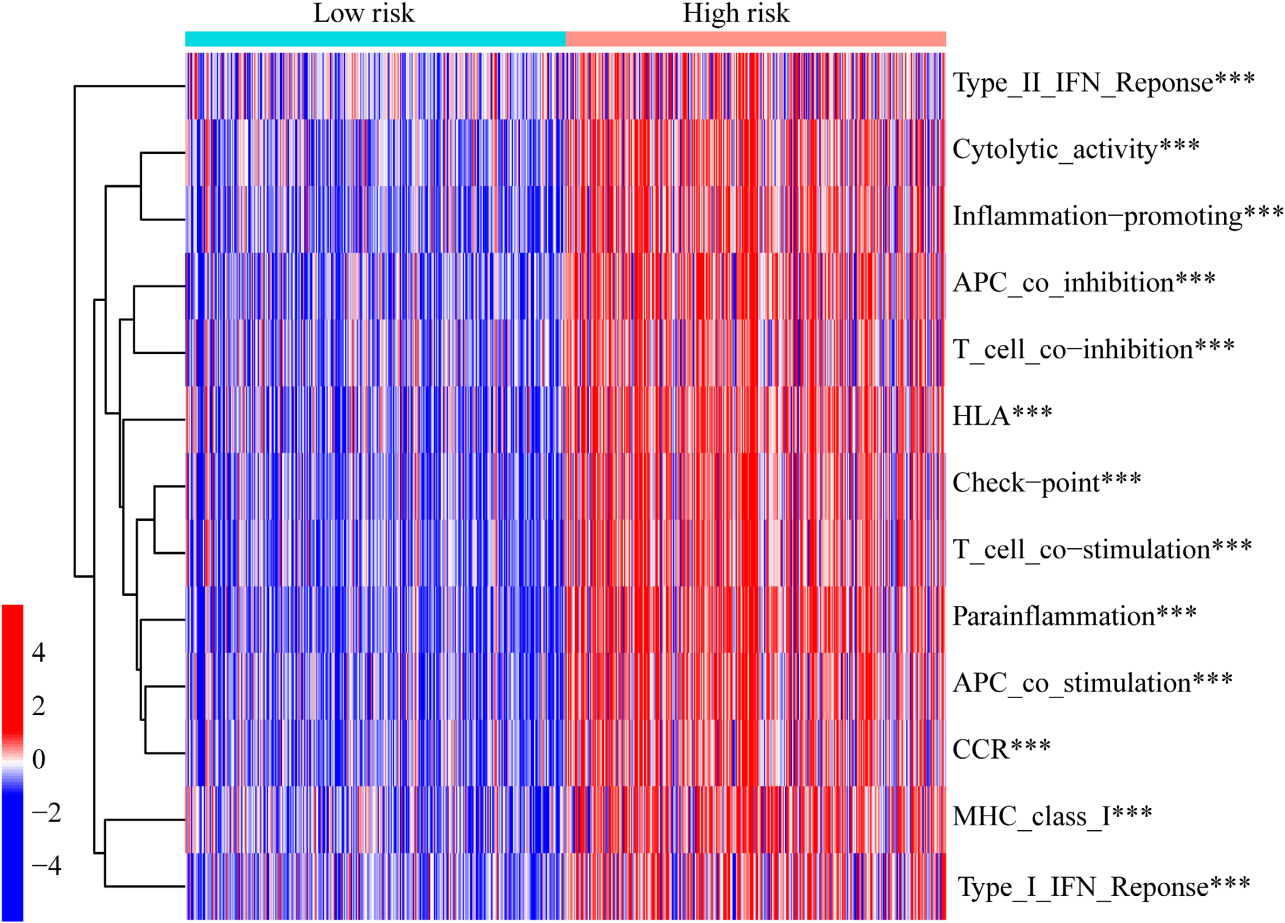
Heatmap showing the differentiated immune pathways between low‐ and high‐risk groups.

The gene variation of the low‐ and high‐risk groups was evaluated in the TCGA cohort. The top 15 genes with the highest alteration frequency between the low‐ and high‐risk groups were shown in Figure 6A,B. The TMB scores indicated that the TMB of the high‐risk group was significantly higher than that of the low‐risk group (*p* < 2.22 e−16, Figure 6C). Thus, the CRlncRNA‐based risk model correlated with TMB. Further, survival analyses revealed that the high‐TMB group had a poorer prognosis than the low‐TMB group (*p* < 0.001, Figure 6D). Furthermore, regardless of the TMB status, the risk level was an effective prognostic factor for glioma patients (*p* < 0.001, Figure 6E). The expression levels of many immune checkpoints in the high‐risk group were significantly higher than those in the low‐risk group in the TCGA cohort (Figure 7), which suggested that glioma patients with high risk might benefit more from Immune Checkpoint Inhibitors (ICI) therapy. Besides, the differentially activated immune pathways between low‐ and high‐risk groups were illustrated as a heatmap, which suggested the low‐ and high‐risk groups had prominent differences in immune pathways including the Type II IFN response, cytolytic activity, inflammation‐promoting, checkpoint inhibition and T‐cell co‐stimulation pathways (*p* < 0.001, Figure 8).

## 4 DISCUSSION

Different from oxidative‐stress‐associated cell death (e.g. apoptosis, necroptosis and ferroptosis), cuproptosis is a unique type of cell death induced by intracellular copper (Cu).^5^ Excess intracellular copper cause the dysfunction of many metabolic enzymes associated with protein lipoylation.^26^ Moreover, lipoylation and Fe‐S cluster proteins are considered targets of copper‐induced toxicity in human cancer cells.^27^ Cuproptosis has attracted a tremendous amount of attention in the field of tumour studies. However, what roles cuproptosis played in glioma and the relationship between cuproptosis‐related lncRNAs and glioma prognosis need to be investigated.

In this study, a prognostic model based on cuproptosis‐related lncRNAs (CRlncRNAs) was constructed by Cox regression and Lasso regression analyses. A TCGA cohort was used as the training group in which 10 CRlncRNAs were selected and included in the risk model construction for glioma patients. In both the training group (TCGA cohort) and the testing group (CGGA cohort), this CRlncRNA‐based risk model showed good prediction for glioma prognosis. Moreover, this model was an independent prognostic factor from conventional clinical and genic features (e.g. grade and age, IDH and MGMT) for glioma patients. The model was superior to that based on conventional clinical features in glioma for prognosis prediction. TMB is based on the somatic mutation number and is associated with neoantigens triggering antitumour immunity.^28^ The TMB has been considered a biomarker to predict prognosis and the response to PD‐L1 treatment for cancers.^29^ High TMB has been approved by the FDA as one of the treatment standards for solid tumour patients to receive ICIs (e.g. a PD‐1 inhibitor, pembrolizumab).^30^ However, many studies have confirmed that there was no association between higher TMB and improved survival in patients with glioma under immunotherapy.^31^, ^32^, ^33^ Therefore, all immune checkpoints in glioma were further analysed. Most of these immune checkpoints, such as PDCD1 (PD‐1), CD274 (PD‐L1), PDCD1L (PD‐L2), CTLA4 and HAVCR2, were significantly different between low‐ and high‐risk groups. Although the treatment efficacies of some ICIs for glioma remain uncertain, many immune checkpoints in our study have no corresponding ICIs until now. Thus ICIs still hold great potentials in glioma therapy. As compared with the low‐risk group, the high‐risk group with higher TMB score and immune checkpoints may not only have more neoantigens for glioma but these patients should also be candidates for immune checkpoint inhibitor therapy. In addition, given the differential expression of immune pathways between low‐ and high‐risk groups, we may reasonably speculate that glioma patients with high‐risk score tend to have more significant immune microenvironment infiltration.

To construct accurate and stable prognostic models for glioma, many researchers have focused their attention on cell‐death‐related genes. Genes associated with ferroptosis, pyroptosis or autophagy have previously been included in the construction of glioma prognostic models, and these models showed good prediction power.^22^, ^34^, ^35^, ^36^ We reasonably speculated that cuproptosis, a recently defined form of cell death, is correlated with glioma prognosis. Although the mechanisms underlying cuproptosis have not been determined, increasing evidence has indicated the significant role played by copper in tumour development and immunity: An increasing number of studies have shown that copper‐induced tumour cell death was associated with mitochondrial dysfunction, proteasome inhibition and antiangiogenesis.^37^, ^38^, ^39^, ^40^ Furthermore, intratumoral copper can regulate key signalling pathways that mediate PD‐L1‐driven cancer immune evasion.^41^ Copper chelators not only increase the levels of tumour‐infiltrating CD8+ T and NK cells but also improve mouse survival.^41^ Our results in this study were consistent with the above research. In our study, we first proposed a prognostic prediction model for glioma based on cuproptosis‐related lncRNAs to facilitate individualized prediction of prognosis, treatment and recurrence. In addition, we further investigated the relationship between cuproptosis in glioma and immunotherapy, which may facilitate individualized treatment strategies. However, there were shortcomings to this study: Firstly, we used TCGA glioma patient data as training data and CGGA as test data. If we could input more distinct data into our model, this would improve the generalization ability of the model. Secondly, this study is only based on bioinformatics analysis and lacks experimental validation. Although the 10 cuproptosis‐related lncRNAs were identified as glioma prognostic markers in this study, more in vitro and in vivo experiments are needed to demonstrate the expression and detailed biological functions of these lncRNAs in glioma. Thirdly, it is crucial to detect the most relevant immune sites and suggest effective treatments.

## 5 CONCLUSION

A novel cuproptosis‐related risk model was constructed for patients with glioma that not only achieved accurate prognostic ability but was also associated with different degrees of immune infiltration. This model provides a theoretical base for the clinical application of cuproptosis‐related lncRNAs on glioma prognosis and immunotherapy. Further studies on the mechanisms underlying cuproptosis will contribute to discovering new treatment targets for glioma.

## AUTHOR CONTRIBUTIONS

**Lin Wang:** Formal analysis (lead); writing – original draft (lead). **Yunqian Li:** Conceptualization (lead); writing – original draft (supporting). **Yubo Wang:** Data curation (equal); investigation (equal). **Jia Li:** Writing – review and editing (supporting). **Yajuan Sun:** Writing – review and editing (supporting). **Jiajun Chen:** Supervision (equal); writing – review and editing (lead). **Ziqian Wang:** Conceptualization (lead); supervision (equal); writing – review and editing (equal).

## FUNDING INFORMATION

This work was supported by National Natural Science Foundation of China [82203647] and Norman Bethune Program of Jilin University [2022B28].

## CONFLICT OF INTEREST

The authors declare that the research was conducted in the absence of any commercial or financial relationships that could be construed as a potential conflict of interest.

## Supporting information


Table S1

## Data Availability

Data available on request from the authors.
