# Supplementary material for: Identification of cuproptosis‐related lncRNAs for prognosis and immunotherapy in glioma
Source: J Cell Mol Med. 2022 Nov 1;26(23):5820–31. doi: 10.1111/jcmm.17603 (PMC9716210; doi:10.1111/jcmm.17603)
Supplement: Supplementary file 1 — Table S1 [file JCMM-26-5820-s001.docx]

|  | TCGA | CGGA 325 | CGGA 693 |
| --- | --- | --- | --- |
| Age | 48.87 | 42.81 | 43.22 |
| Female | 276 | 122 | 290 |
| Male | 376 | 203 | 393 |
| OS | 838 | 1397 | 1137 |
| **Living status** |  |  |  |
| Alive | 404 | 96 | 266 |
| Dead | 248 | 220 | 397 |
| **WHO grade** |  |  |  |
| WHO II | 240 | 103 | 188 |
| WHO III | 255 | 79 | 255 |
| WHO IV | 157 | 139 | 249 |
